# Transient transfection of human *CDNF* gene reduces the 6-hydroxydopamine-induced neuroinflammation in the rat substantia nigra

**DOI:** 10.1186/s12974-014-0209-0

**Published:** 2014-12-16

**Authors:** Rasajna Nadella, Merja H Voutilainen, Mart Saarma, Juan A Gonzalez-Barrios, Bertha A Leon-Chavez, Judith M Dueñas Jiménez, Sergio H Dueñas Jiménez, Lourdes Escobedo, Daniel Martinez-Fong

**Affiliations:** Programa de Doctorado en Nanociencias y Nanotecnología; CINVESTAV, Av. Instituto Politécnico Nacional # 2508, San Pedro Zacatenco, CP 07360 México, DF México; Departamento de Fisiología, Biofísica y Neurociencias; CINVESTAV, Av. Instituto Politécnico Nacional # 2508, San Pedro Zacatenco, CP 07360 México, DF México; Institute of Biotechnology, PO Box 56, Viikki Biocenter, University of Helsinki, FI-00014 Helsinki, Finland; Laboratorio de Medicina Genómica, Hospital Regional ‘1° de Octubre’, ISSSTE, Av. Instituto Politécnico Nacional # 1667, Magdalena de las Salinas, CP 02800 México, DF México; Facultad de Ciencias Químicas, Benemérita Universidad Autónoma de Puebla, Avenida San Claudio S/N, Ciudad Universitaria Edif. 105A, CP 72570 Puebla, PUE México; Laboratorio de Neurofisiología, Departamento de Fisiología, Centro Universitario de Ciencias de la Salud, Universidad de Guadalajara, Av. Juárez 976, Colonia Centro, CP 44100 Guadalajara, Jalisco México; Departamento de Neurociencias, Centro Universitario de Ciencias de la Salud, Universidad de Guadalajara, Av. Juárez 976, Colonia Centro, CP 44100 Guadalajara, Jalisco México

**Keywords:** NG2 cells, Astrocytes, NBRE promoter, Neurotrophic factor, CDNF, Polyplex, Cytokines, Nitrosative stress

## Abstract

**Background:**

The anti-inflammatory effect of the cerebral dopamine neurotrophic factor (CDNF) was shown recently in primary glial cell cultures, yet such effect remains unknown both *in vivo* and in 6-hydroxydopamine (6-OHDA) models of Parkinson’s disease (PD). We addressed this issue by performing an intranigral transfection of the human *CDNF* (hCDNF) gene in the critical period of inflammation after a single intrastriatal 6-OHDA injection in the rat.

**Methods:**

At day 15 after lesion, the plasmids p3xNBRE-hCDNF or p3xNBRE-EGFP, coding for enhanced green florescent protein (EGFP), were transfected into the rat substantia nigra (SN) using neurotensin (NTS)-polyplex. At day 15 post-transfection, we measured nitrite and lipoperoxide levels in the SN. We used ELISA to quantify the levels of TNF-α, IL-1β, IL-6, endogenous rat CDNF (rCDNF) and hCDNF. We also used qRT-PCR to measure rCDNF and hCDNF transcripts, and immunofluorescence assays to evaluate iNOS, CDNF and glial cells (microglia, astrocytes and Neuron/Glial type 2 (NG2) cells). Intact SNs were additional controls.

**Results:**

In the SN, 6-OHDA triggered nitrosative stress, increased inflammatory cytokines levels, and activated the multipotent progenitor NG2 cells, which convert into astrocytes to produce rCDNF. In comparison with the hemiparkinsonian rats that were transfected with the EGFP gene or without transfection, 6-OHDA treatment and p3xNBRE-hCDNF transfection increased the conversion of NG2 cells into astrocytes resulting in 4-fold increase in the rCDNF protein levels. The overexpressed CDNF reduced nitrosative stress, glial markers and IL-6 levels in the SN, but not TNF-α and IL-1β levels.

**Conclusion:**

Our results show the anti-inflammatory effect of CDNF in a 6-OHDA rat of Parkinson’s disease. Our results also suggest the possible participation of TNF-α, IL-1β and IL-6 in rCDNF production by astrocytes, supporting their anti-inflammatory role.

**Electronic supplementary material:**

The online version of this article (doi:10.1186/s12974-014-0209-0) contains supplementary material, which is available to authorized users.

## Background

Cerebral dopamine neurotrophic factor (CDNF) and mesencephalic astrocyte-derived neurotrophic factor (MANF) are evolutionarily-conserved proteins that form the new family of neurotrophic factors with unique structure [1-8]. The main distinguishing feature of this family is that the members (CDNF and MANF) contain eight cysteine residues that were conserved in evolution from invertebrates to vertebrates [6,7]. CDNF was first identified by bioinformatics tools and was then characterized biochemically [2]. Pre-CDNF protein is 187 amino acids long whereas mature CDNF protein (GenBank access: NM_177647) comprises 161 amino acid residues with the molecular weight of 18 kDa. The mature CDNF protein consists of an N-terminal saposin-like domain that may bind lipids and a C-terminal domain that is structurally similar to members of the SAP protein superfamily [9]. Although CDNF and MANF are secreted proteins, they contain an endoplasmic reticulum (ER) retention signal [6,8] and are, therefore, located mainly in the ER. Neuroprotective and neurorestorative effects of striatal CDNF injections were demonstrated in 1-methyl-4-phenyl-1,2,3,6-tetrahydropyridine (MPTP)-treated mice [10]. In 6-hydroxydopamine (6-OHDA)-induced parkinsonian rat models, intrastriatal *CDNF* gene therapy mediated by viral vectors [11,12] and intrastriatal injection of CDNF protein as a single dose [2] or chronic infusion [13] also showed protective effects. *CDNF* gene therapy has shown a regenerative effect in the rat sciatic nerve injury model [14]. In addition to the neurotrophic effects, recent studies on primary glial cell cultures suggest that CDNF has an anti-inflammatory effect [15,16]. However, it remains unknown whether CDNF is able to revert from neuroinflammation *in vivo* and in an animal model of Parkinson’s disease (PD).

Clinical, experimental, epidemiological, and pathological evidence indicates that neuroinflammation plays an important role in PD [17-22]. *Postmortem* studies have shown the presence of activated microglia and reactive astrocytes [17-22]. Accordingly, increased levels of nitric oxide (NO), inducible nitric oxide synthase (iNOS), cyclooxygenase 2 (COX2) and pro-inflammatory cytokines have been found in the brains of PD patients [23]. A similar inflammatory pattern has been found in the rat SN after the striatal injection of 6-OHDA [24-29]. In this model, the role of the increased levels of pro-inflammatory cytokines is controversial because recent studies have shown that those cytokines in the neurodegenerative process can exert protective effects acting as neurotrophic factors [30-32].

After injury, mature astrocytes can proliferate and acquire stem cell properties suggesting their capacity to promote neuronal regeneration [33-35]. Other cells that have been involved in neuronal regeneration are precursor cells called Neuron/Glial type 2 (NG2) glia. These NG2 glia are multipotent progenitor cells [36,37] with neurogenic [38], oligodendrogenic [39,40], astrogenic [41,42] and microgliogenic [43] properties. However, the role of NG2 cells has not been fully examined in the 6-OHDA PD rat model. A recent study has only shown that an intrastriatal 6-OHDA injection stimulates the conversion of NG2 cells into microglia, which produce GDNF, a neurotrophic factor for dopaminergic neurons [43]. However, conversion ability of NG2 cells to produce astrocytes that lead to the production of neurotrophic factors in PD and animal models of this disease remains unclear.

Neurotensin (NTS)-polyplex is a synthetic nanocarrier that enables gene delivery via internalization of NTS receptor type 1 into dopaminergic neurons in the substantia nigra (SN) and in primary cultures [44-48]. Some studies have shown that NTS-polyplex is an efficient tool to transfer neurotrophic factor genes such as *GDNF*, *BDNF* or Neurturin (*NRTN*) into dopaminergic neurons after inducing lesion by an intrastriatal 6-OHDA injection [44,49]. In these models of neurotrophic factor gene therapy, the transgene expression has been correlated with a decrease of motor impairment, increase in DA levels and recovery of nigrostriatal dopaminergic system from a neurodegeneration [44,49]. The safety of NTS-polyplex-mediated transfection is an added advantage [50]. A recent work has shown that a 15-day period following the single intrastriatal injection of 6-OHDA is very critical for the development of apoptosis and glia activation in the SN [51]. Based on all the antecedents, we hypothesized that the intranigral transfection of the human *CDNF* (hCDNF) gene in the 6-OHDA PD animal model would decrease neuroinflammation and shows the participation of NG2 cells in overexpression of CDNF. The hCDNF gene transfection was attained using NTS-polyplex in the SN at day 15 after the intrastriatal injection of 6-OHDA and, at day 15 post-transfection, nitrosative/oxidative stress markers, pro-inflammatory cytokines (TNF-α, IL-1β and IL-6), glial activation, endogenous and transgenic CDNF levels were determined in the SN of male Wistar rats. Our work uncovers the *in vivo* anti-inflammatory role of CDNF during the critical period for dopaminergic neuronal survival in an animal model of PD.

## Methods

### Plasmids

Plasmids p3xNBRE-hCDNF (4,482 bp) coding for hCDNF and p3xNBRE-EGFP (4,684 bp) coding for EGFP (enhanced green florescent protein), a reporter gene, were obtained by cloning the human CDNF cDNA (GenBank access: NM 001029954) or the EGFP cDNA (GenBank access: U55762) under the 3xNBRE (a synthetic and tissue specific promoter based on Nurr1 element response) promoter in the plasmid pGL2 basic 3xNBRE-LUC. LUC cDNA was removed by *Hind* III - *PflM* I restriction enzymes (REs) from the pGL2 basic 3xNBRE-LUC, whereas hCDNF cDNA (560 bp) was excised by *Hind* III - *Eco*R V REs from pCR3.1-hCDNF and EGFP cDNA (716 bp) by *Hind* III - *Not* I REs from pEGFP-N1 (Clontech; Mountain View, CA, USA). The cohesive ends resulting from *PflM* I and *Not* I digestion were treated with Klenow fragment (Thermo Scientific; Mexico City, Mexico) to convert them into blunt ends. hCDNF cDNA or EGFP cDNA were cloned in the sense orientation into the *Hind* III - *PflM* I sites in the plasmid pGL2 basic 3xNBRE-LUC, replacing *LUC* gene. Restriction digestion and automatic DNA sequencing confirmed full hCDNF and EGFP sequences and their correct orientation in the plasmids. Transfections with pCR3.1-hCDNF, encoding hCDNF gene under CMV (cytomegalovirus) promoter, and p3xNBRE-EGFP acted as a positive and negative controls respectively.

### Synthesis of the neurotensin carrier and polyplexes

The detailed procedure of NTS carrier synthesis and of NTS-polyplex formation at an optimum molar ratio and its biophysical characteristics were reported previously [45,47,48]. Briefly, NTS (Sigma-Aldrich; St Louis, MO, USA) and FP (GLFEAIAEFIEGGWEGLIEGCAKKK; purity > 90%; SynPep Corp.; Dublin, CA, USA) were cross-linked with poly-L-lysine (48 kDa mean molecular mass; Sigma-Aldrich; St Louis, MO, USA) using LC-SPDP (Thermo Scientific Pierce; Rockford, IL, USA) as the cross-linker [45]. Suitable gel-filtration chromatography was used to purify the SPDP-derivatives and the NTS-SPDP-(FP-SPDP)-poly-L-lysine conjugate, the NTS carrier. This conjugate was concentrated to 1 mL, further dialyzed against PBS solution, pH 7.4, and sterilized by filtration. The NTS-polyplexes were made by electrostatically binding the mutant Vp1 SV40 KP (MAPTKRKGSCPGAAPNKPK; 90% purity; SynPep Corp., Dublin, CA, USA) to pDNA [45,47,52]. Retardation and retention gel assays were used to determine and calculate the optimal molar ratio of polyplex components as described in detail elsewhere [45,47,48,52,53]. Accordingly, the final optimum molar ratio of NTS-polyplex components for all the plasmids used were 30 nM pDNA: 36 μM KP: 900 nM NTS-carrier and, at this molar ratio, the concentration of NTS used was 385.6 pmol/μL, calculated as per ^125^I-NTS [47,50]. Based on the concentration and size of pDNAs, the dose of pDNA injected was 88 ng/μL of p3xNBRE-hCDNF (4,482 bp), 111 ng/μL of pCR3.1-hCDNF (5,624 bp) and 92.7 ng/μL of p3xNBRE-EGFP (4,684 bp).

### Intracranial injections

Male Wistar rats (200 to 230 g) housed together (5 per cage) with constant temperature (23°C), water and food available *ad libitum* and kept under natural light-dark cycle (12-12 hours) were used. Rats were anesthetized with ketamine/xilazine (100 mg/kg/60 mg/kg intraperitoneally) and placed on a stereotaxic frame (Stoelting; Wood Dale, IL, USA). The hemiparkinsonism was generated by injecting single dose of 6-OHDA solution (Sigma-Aldrich; St Louis, MO, USA) (20 μg/3 μL of PBS containing 0.2% ascorbic acid) into the left striatum at the following coordinates: AP +6.9 mm from interaural midpoint, ML +4.0 mm from intraparietal suture, DV −5.5 mm from dura mater. The flow rate of injection was 0.2 μL/minute [51].

**Figure 1:**
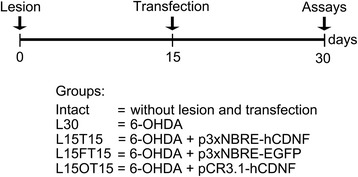
**Experimental design.** At day 0, we injected 6-hydroxydopamine (6-OHDA) into the striatum; at day 15 post-lesion, we transfected either of the mentioned plasmids with neurotensin (NTS)-polyplex into the substantia nigra pars compacta and at day 30 from the beginning of the experiment or at day 15 after the transfection, we extracted the tissues or perfused the animals for molecular and cellular assays.

NTS-polyplex harboring the plasmid p3xNBRE-hCDNF was injected at day 15 after the lesion with 6-OHDA (referred as L15T15; Figure 1) because the maximum peak of apoptosis and activated glial cells was detected at that day [51]. The injection was made into the ipsilateral SN at the following coordinates: AP, +2.4 mm from interaural midpoint; ML, +1.8 mm from intraparietal suture, DV −6.9 mm from dura mater. Three microliters of NTS-polyplex solution were injected at a flow rate of 0.1 μL/minute. NTS-polyplex harboring the plasmids p3xNBRE-EGFP, labeled as L15FT15 (Figure 1), and pCR3.1-hCDNF, labeled as L15OT15 (Figure 1), were also injected to maintain negative and positive controls respectively. Detailed experimental scheme was shown in Figure 1.

### qRT-PCR (quantitative Reverse TranscriptionPolymerase Chain Reaction)

Total RNA was isolated from the brain tissues (striatum and SN) using Trizol (Invitrogen Corporation, Carlsbad, CA, USA) and then RNA preparations were treated with RNase-free DNase I. Reverse transcription was made with 750 ng of total RNA for striatum and 250 ng of total RNA for SN with SuperScript III reverse transcriptase (200 U) using 0.1 mg of oligo dT (Invitrogen Corporation, Carlsbad, CA, USA). The reverse transcribed product was diluted 4 times with molecular biology grade water and 2.5 μL of this diluted cDNA was mixed with 2× TaqMan Universal Mastermix and 20× TaqMan gene specific probe (Applied Biosystems; Foster City, CA, USA) in a final volume of 5 μL. cDNAs were amplified for 45 cycles using a 7900HT Fast Real Time PCR system (Applied Biosystems; Foster City, CA, USA). TaqMan gene specific probes used for experiments were: Rn00667869_m1 for rat β-actin; Rn01525859_g1 for rat TNF-α; Rn00580432_m1 for rat IL-1β; Rn01410330_m1 for rat IL-6; Rn01765001_m1 for rat CDNF and Hs00418490_m1 for human CDNF (Applied Biosystems; Foster City, CA, USA). β-Actin mRNA levels were also amplified for each sample and used as internal control and for normalization. The cycle threshold (Ct) values for β-actin, rCDNF and hCDNF were measured and calculated by Sequence Detection System software (SDS 2.2; Applied Biosystems; Foster City, CA, USA). Relative transcript levels were expressed as the fold-change for gene expression and were calculated using the 2^-∆∆Ct^ method [54,55]. For the untreated control samples, ΔΔC_t_ equals 0 and 2^0^ is 1, the fold change in rCDNFgene expression in intact condition is 1 [56].

### ELISA

Homogenization of brain tissues (striatum and SN) was made using protein extraction buffer containing 100 mM Tris HCl (pH 7.4), 750 mM NaCl (sodium chloride), 10 mM EDTA (ethylenediaminetetraacetic acid), 5 mM EGTA (ethylene glycoltetraacetic acid) and a cocktail of protease inhibitors (Roche, Basel, Switzerland) [57]. The samples were centrifuged at 1,000 g for 10 minutes at 4°C and then supernatant was centrifuged again at 20,000 g for 40 minutes at 4°C to remove the remaining debris. For detecting the levels of inflammatory cytokines, indirect ELISA was done as per the user guide using Milliplex MAP Rat cytokine/chemokine magnetic bead panel kit (RECYTMAG_65K; Millipore; Temecula, CA, USA) and reading was done by LUMINEX MAGPIX® detection system with xPONET software (Millipore Corporation; Billerica, MA, USA). Sensitivity of TNF-α ranges between 1.9 to 156.3 pg/mL, of IL-1β ranges between 2.8 to 2,500.0 pg/mL and of IL-6 ranges between 30.7 to 4,687.5 pg/mL. For detecting the levels of hCDNF and rCDNF, Sandwich enzyme immunoassay was done using separate kits (SEG458Hu and SEG458Ra; Uscn Life Science Inc; Wuhan, Hubei, China) and absorbance was read at 450 nm using a Multiskan® MULTISOFT spectrophotometer (Labsystems; Tokyo, Japan).

### Immunofluorescence

Serial coronal sections of 30-μm thickness were cut from perfused and fixed rat brains with 4% paraformaldehyde. Before starting the experiment, the slices were rinsed once with PBS 1× for 5 minutes. To increase the permeability of reagents into the tissue, the slices were incubated with PBS-Triton 0.1% for 20 minutes and then by PBS-SDS 0.5% for 5 minutes. These permeabilized sections were incubated with 1% BSA in PBS-Triton 0.1% for 30 minutes to block the unspecific binding sites. Immediately after blocking, primary antibody solution was added to the tissue slices and was incubated overnight at room temperature on a rotary shaker. This is followed by addition of secondary antibody and incubated for 2 hours at room temperature on a rotary shaker. Between each step, washing with PBS (1x)was done × 3 for 5 minutes each.

For all double and triple immunofluorescence experiments, primary antibodies used were mouse monoclonal anti-tyrosine hydroxylase (TH), clone TH-2, ascites fluid (1:1,000; Sigma-Aldrich; St Louis, MO, USA), rabbit polyclonal anti-TH (1:1,000; Chemicon Inc.; Billerica, MA, USA), rabbit polyclonal anti-CDNF (1:1,000; ProSci Inc; San Diego, CA, USA), mouse monoclonal anti-iNOS (1:1,000; BD Transduction Laboratories; BD Biosciences; San Jose, CA, USA), mouse monoclonal anti-CD11b (OX42), a marker for microglia (1:200; Abcam; Cambridge, UK), rabbit polyclonal anti-OX42 (1:250; Abcam; Cambridge, UK), goat polyclonal anti-OX42 (1:300; Abcam; Cambridge, UK), mouse monoclonal anti-glial fibrillary acidic protein (GFAP) (1:250; Cell Signaling; Danvers, MA, USA), rabbit polyclonal anti-GFAP (1:300; Cell Signaling; Danvers, MA, USA), goat polyclonal anti-GFAP (1:300; Cell Signaling; Danvers, MA, USA), mouse monoclonal anti-NG2 (1:1,000; Upstate Cell Signaling solutions; Temecula, CA, USA) and rabbit polyclonal anti-NG2 (1:300; Millipore; Temecula, CA, USA). Irrelevant antibody of the same IgG subclass was added in place of primary antibody for all the negative controls.

The secondary antibodies used were Texas red anti-mouse IgG (H + L) made in horse (1:900; Vector Laboratories; Burlingame, CA, USA), Texas red anti-rabbit IgG (H + L) made in goat (1:400; Vector Laboratories; Burlingame, CA, USA), Texas red anti-goat IgG (H + L) made in donkey (1:500; Vector Laboratories; Burlingame, CA, USA), Alexa Fluor 488 anti-mouse IgG (H + L) made in chicken (1:200; Invitrogen Molecular Probes; Eugene, OR, USA), Alexa Fluor 488 anti-rabbit IgG (H + L) made in chicken (1:200; Invitrogen Molecular Probes; Eugene, OR, USA), Alexa Fluor 488 anti-goat IgG (H + L) made in donkey (1:500; Invitrogen Molecular Probes; Eugene, OR, USA), Cy3 anti-mouse IgG (H + L) made in goat (1:300; Invitrogen Molecular Probes; Eugene, OR, USA).

Fluorescence labeling was viewed through a multispectral confocal laser-scanning microscope (TCS-SPE, Leica; Heidelberg, Germany) using 40× and 63× oil-immersion objectives at excitation-emission wavelengths of 405 to 465 nm (blue channel), 488 to 522 nm (green channel), and 568 to 635 nm (red channel). Their consecutive 1-μm optical sections were also obtained in the z-series (scanning rate 600 Hz). The images were acquired using LAS AF software (Leica application suite; Leica Microsystems, Nussloch, Germany). Quantification was made using the same LAS AF software by placing a rectangular region of interest (ROI) across the full image and within the ROI, for every image, mean fluorescence intensity (MFI) was measured and the values plotted.

### Nitrite assay

The content of nitric oxide (NO) was determined by measuring the accumulation of nitrites (NO_2_^−^) in the supernatant of homogenized SN samples of rat brain using the colorimetric method as described elsewhere [58]. Briefly, tissue samples from SN were mechanically homogenized in PBS and centrifuged at 12,500 rpm for 30 minutes at 4°C by using 17TR microfuge (Hanil Science Industrial Co. Ltd; Inchun, Korea). Nitrite concentration in 100 μL of supernatant was measured by the colorimetric reaction generated by the addition of 100 μL of Griess reagent (composes equal volumes of 0.1% N-(1-naphthyl) ethylenediamine dihydrochloride and 1.32% sulfanilamide in 60% acetic acid). The absorbance of the samples was determined at 540 nm with a SmartSpec 3000 spectrophotometer (Bio-Rad; Hercules, CA, USA) and interpolated by using a standard curve of sodium nitrite (NaNO_2_; 1 to 10 μM) to calculate the nitrite content.

### Lipoperoxide assay

Assay of lipoperoxide was made by measuring malondialdehyde (MDA) and 4-hydroxyalkanal (4-HEA) in the supernatant of homogenized SN samples of rat brain using the colorimetric method [58]. Briefly, the colorimetric reaction in 200 μL of supernatant was produced by subsequent addition of 0.65 mL of 10.3 mM N-methyl 2-phenylindole diluted in a mixture of acetonitrile:methanol (3:1). The reaction was initiated by the addition of 150 μL of methanesulfonic acid. This reaction mixture was strongly agitated and incubated at 45°C for 1 hour and then centrifuged at 3,000 rpm for 10 minutes. The absorbance of supernatant was read at 586 nm with a SmartSpec 3000 spectrophotometer (Bio-Rad; Hercules, CA, USA). The absorbance values were compared to a standard curve in the concentration range of 0.5 to 5 μM of 1,1,3,3-tetramethoxypropane (10 mM stock) to calculate the MDA and 4-HEA contents in the samples.

### Ethics statement

All procedures were in accordance with the current Mexican legislation, NOM-062-ZOO-1999 (SAGARPA), based on the Guide for the Care and Use of Laboratory animals, NRC. CINVESTAV Institutional Animal Care and Use Committee approved our procedures for animal use (protocol # 109-02). All efforts were made to minimize animal suffering.

### Statistical analysis

All results were expressed as mean ± standard deviation (SD) values, obtained at least from 4 independent experiments (n = 4). The comparison among the groups was made using one-way analysis of variance (ANOVA) followed by the Newman-Keuls *post hoc* test using the GraphPad Prism 5.0 (GraphPad Software Inc., La Jolla, CA, USA). Accepted significance was at *P* < 0.05.

## Results

### Pattern and levels of total CDNF expression in the SN

**Figure 2:**
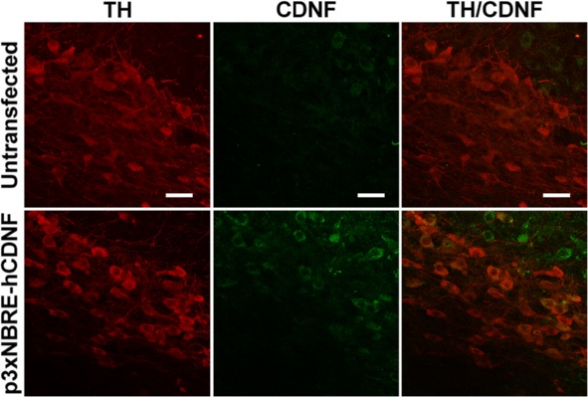
**Presence of total cerebral dopamine neurotrophic factor (CDNF) + cells in intact rat substantia nigra (SN).** Representative confocal micrographs showing the immunoreactivity to tyrosine hydroxylase (TH), the rate-limiting enzyme in dopamine synthesis (red) and CDNF (green). TH/CDNF is the overlay of the micrographs in the same row. Untransfected and p3xNBRE-hCDNF transfected tissue slices were from different intact rat brains. The scale bars = 20 μm are common for all the micrographs.

The immunofluorescence studies in the untransfected SN of intact rats showed that CDNF+ cells do not co-localize with cells immunoreactive to TH, the rate-limiting enzyme in dopamine synthesis (Figure 2, top row), but largely co-localize with TH+ cells 7 days after p3xNBRE-hCDNF transfection (Figure 2, bottom row). These results show the feasibility of targeted hCDNF gene delivery (Figure 2). As the endogenous presence of rat CDNF (rCDNF) was found in an intact rat brain, it was very important to explore whether the 6-OHDA lesion and the hCDNF transfection influence the endogenous rCDNF expression levels. As the antibody can equally detect both rCDNF and hCDNF, we used species-specific probes and kits for qRT-PCR and ELISA assays respectively for the experimental groups.

**Figure 3:**
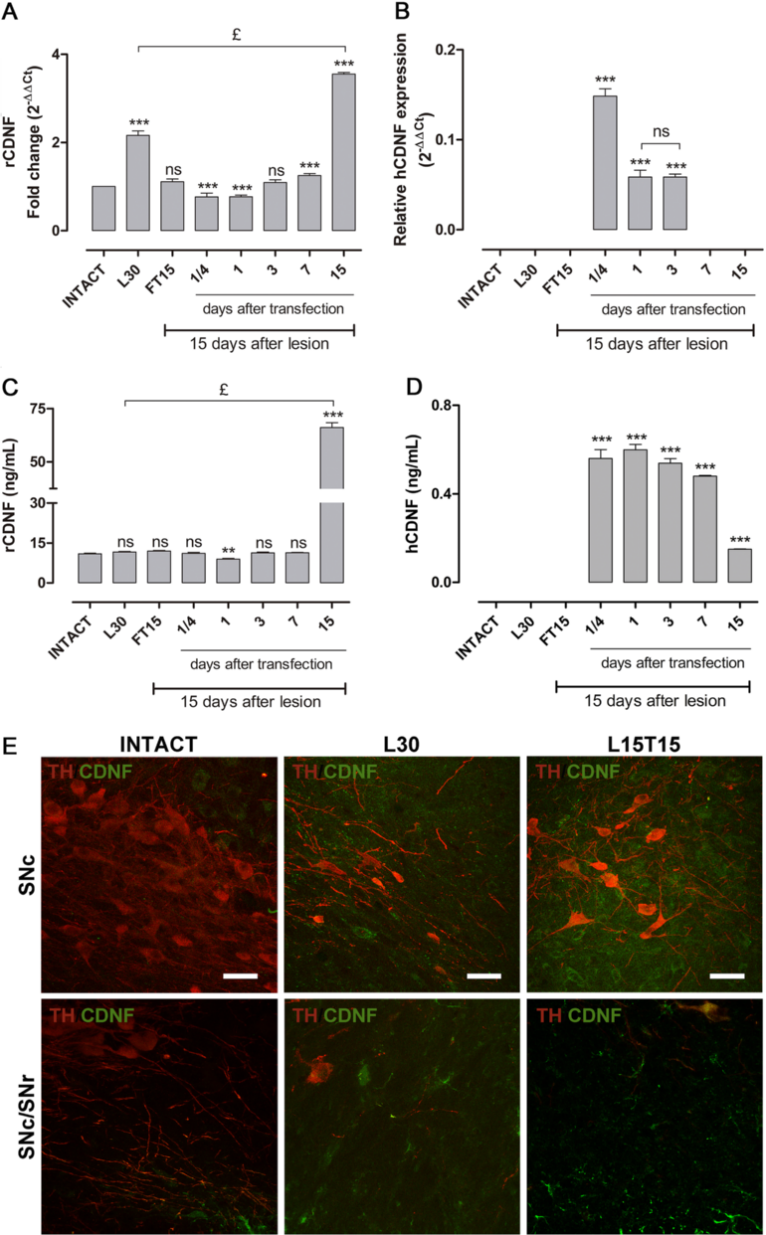
**Expression levels of total cerebral dopamine neurotrophic factor (CDNF) in the substantia nigra (SN).** Graphs showing the fold change increase in the endogenous, rat (r)CDNF **(A)** and relative transgene human (h)CDNF **(B)** transcripts by qRT-PCR. Graphs showing the protein levels of rCDNF **(C)** and hCDNF **(D)** by ELISA. First 3 bars in the graphs represent controls and the rest represent the time course of p3xNBRE-hCDNF transfections. FT15 = at day 15 after p3xNBRE-EGFP transfection, ¼ = at 6 hours; 1 = at 24 hours; 3 = at day 3; 7 = at day 7; 15 = day 15, after p3xNBRE-hCDNF transfection in a 15-day lesion rats. **(E)** Representative merged confocal micrographs showing immunoreactivity to CDNF (green) and tyrosine hydroxylase (TH) (red). The headings refer to mesencephalon slices that were obtained from different rats, intact (INTACT), with 30-day 6-OHDA lesion (L30), and 15 days of lesion and 15 days of transfection (L15T15). The scale bars = 20 μm are common for all the micrographs. ns = not significant. **P < 0.01, ***P < 0.001 when compared with the intact condition. £ = *P* < 0.001 when compared with L30. One-way ANOVA with Newman-Keuls *post hoc* test. n = 4 different rats for each condition.

qRT-PCR assays in the SN at day 30 after lesion (L30) showed 2-fold increase (2.162 ± 0.103) in rCDNF transcripts than basal levels in intact condition. At this time, an additional increase (3.559 ± 0.037) in 6-OHDA-induced levels of rCDNF transcripts in the SN was found at day 15 after transfection with p3xNBRE-hCDNF (L15T15), but not (1.107 ± 0.066) with a reporter gene (L15FT15**)** transfection (Figure 3A). Surprisingly, the qRT-PCR assays showed that hCDNF mRNA expression was transient, when transfected at day 15 after 6-OHDA lesion (Figure 3B). High levels of hCDNF transcripts were measured at 6 hours (0.149 ± 0.008) after transfection, and these levels reduced almost to half after 24 hours (0.058 ± 0.008) and were maintained until 3 days (0.058 ± 0.004) after transfection to disappear thereafter (Figure 3, B). At the same time, the highest hCDNF protein levels were measured at 6 hours (0.56 ± 0.04 ng/mL) and at 24 hours (0.599 ± 0.024 ng/mL) after transfection, but the levels started to decrease at day 3 (0.538 ± 0.021 ng/mL) and further decreased until L15T15 (0.150 ± 0.001 ng/mL) (Figure 3D). However, no significant change in rCDNF protein levels was found among intact (10.97 ± 0.19 ng/mL), L30 (11.63 ± 0.19 ng/mL) and L15FT15 (11.95 ± 0.29 ng/mL) conditions. A 6-fold increase in rCDNF protein levels was detected in L15T15 (66.08 ± 2.29 ng/mL), when compared to those in intact condition (Figure 3C). Together, these results suggest that the transient transgene expression triggered the expression of endogenous rCDNF.

Double immunofluorescence studies showed that CDNF immunoreactivity exhibits different cellular location in the SN under different experimental conditions. In the SN pars compacta (SNc) of an intact rat, CDNF+ cells were absent and instead they are present in the parabranchial pigmented (PBP) nucleus, a nucleus in medial ventral tegmental area that borders SNc ventrally (Figure 3E, top row). In L30 (Figure 3E top row) and L15T15 (Figure 3E, top row) conditions, CDNF immunoreactivity is present in and around TH+ cells in the SNc. In the SNc/SN reticulata (SNr) inter-region, CDNF immunoreactivity is absent in intact rats but present in L30 and L15T15 (Figure 3E, bottom row). Especially in this inter-region of SNc/SNr, the morphology of CDNF+ cells was suggestive of glial cells and their branches are more notable in L15T15 but less evident in L30 (Figure 3E, bottom row).

### Activation and conversion of NG2 cells into astrocytes that produce CDNF in the SNr

**Figure 4:**
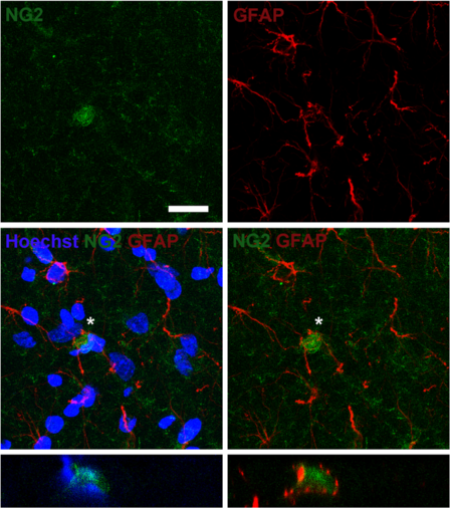
**Apparent conversion of NG2 cell into an astrocyte cell in the substantia nigra pars reticulata (SNr) at day 30 after 6-hydroxydopamine (6-ODHA) lesion.** Representative confocal micrographs showing nuclei stained with Hoechst (blue) and immunostaining against NG2 (green), and GFAP (red). Individual micrographs of NG2 and GFAP (top row), merged images of Hoechst/NG2/GFAP and NG2/GFAP (middle row) and vertical 1-μm optical slices for the cell indicated by the asterisk (bottom row) are shown. The scale bar = 20 μm is common for all the micrographs. GFAP, glial fibrillary acidic protein; NG2, Neuron/Glial type 2.

**Figure 5:**
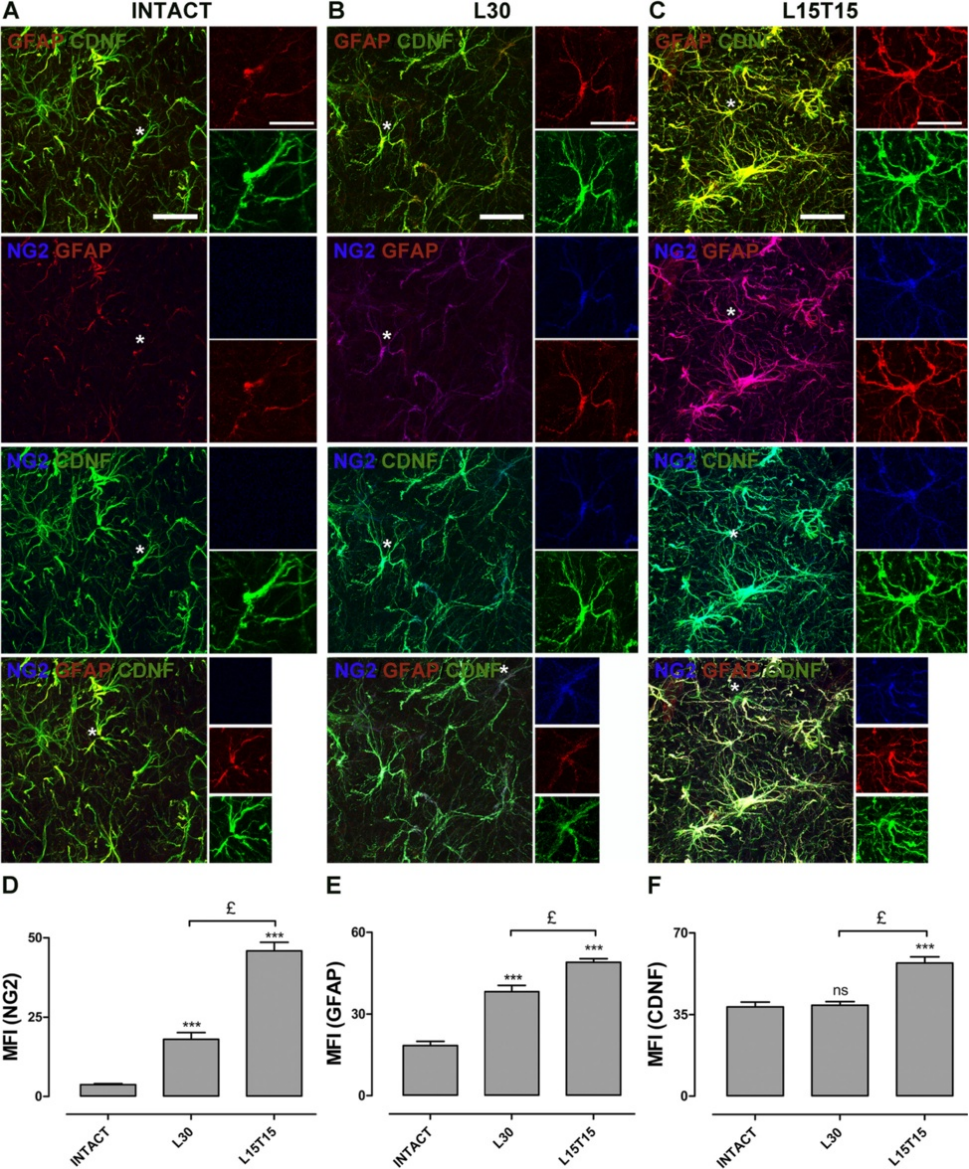
**Expression of cerebral dopamine neurotrophic factor (CDNF) by astrocytes and astrocyte-like NG2 cells in the substantia nigra pars reticulata (SNr).** Representative merged confocal micrographs showing immunoreactivity to CDNF (green), GFAP (red) and NG2 (blue). The triple immune micrographs in the lower row are shown individually in the first three rows with possible combinations of double markers to see more details. The headings refer to mesencephalon slices that were obtained from different rats, intact **(A)**, with 30-day 6-OHDA lesion **(B)**, and 15 days of lesion and 15 days of transfection **(C)**. Asterisk represents the cell whose fluorescent markers are individually displayed in the inserts. Graphs showing the mean fluorescence intensity (MFI) for NG2 **(D)**, GFAP **(E)** and CDNF **(F)**. The scale bars = 20 μm are common for all the micrographs. ns = not significant. ***P < 0.001 when compared with the intact condition. £ = *P* < 0.001 when compared with L30. One-way ANOVA with Newman-Keuls *post hoc* test. n = 4 different rats for each condition. GFAP, glial fibrillary acidic protein; NG2, Neuron/Glial type 2.

6-OHDA toxicity is known to activate various glial cell types [51]. Accordingly, confocal analysis showed the presence of NG2+ cells (Figure 4, NG2) and GFAP+ cells (Figure 3, GFAP) in the SNr 30 days after lesion (L30), and co-localization of these cells as shown by the merged images (Figure 4, middle row) and by z optical sections (Figure 4, bottom row). In intact SNr, NG2+ cells and NG2/GFAP double positive cells were absent and only a basal number of GFAP+ cells were found (Figure 5A). These results suggest that the 6-OHDA lesion induced the conversion of NG2 cells into astrocyte cells (Additional file 1: Figure S1).

We further explored whether NG2/GFAP double positive cells might produce CDNF. Remarkably, rCDNF is produced by astrocytes in intact rat SNr (Figure 5A). In L30, rCDNF+ cells were also NG2/GFAP double positive cells (Figure 5B). Interestingly, in L15T15, there was profound branching of NG2/GFAP/CDNF triple positive cells (Figure 5C), thus suggesting that transfection of hCDNF leads to conversion of more NG2 cells to astrocytes, which lead to increased production of rCDNF (Figure 3A and Figure 3C). Mean fluorescence intensity (MFI) for NG2 (Figure 5D) showed 5-fold increase with 6-OHDA lesion in L30 (18.05 ± 2.12) when compared to that of intact condition (3.73 ± 0.31), and further 2-fold increase with hCDNF transfection (45.85 ± 2.74) when compared to that of L30. MFI for GFAP (Figure 5E) increased double (38.3 ± 2.3) with lesion and almost triple (48.97 ± 1.36) with transfection when compared to that of basal levels in the intact condition (18.42 ± 1.52). Whereas, MFI for CDNF (Figure 5F) showed no significance in L30 (39.04 ± 1.57) but increased almost double in L15T15 (57.1 ± 2.6), when compared to intact condition (38.37 ± 2.05), supporting the ELISA results (Figure 3C). On the other hand, neither this phenomenon of conversion of NG2 cells to microglia to produce CDNF, nor NG2/OX42/CDNF triple positive cells were found at L15T15 (Additional file 2: Figure S2).

In addition, we explored at day 15 after transfection whether this production of CDNF by astrocytes and astrocyte-like NG2 cells depends on transgene or promoter. Transfection of hCDNF under CMV, a non-specific promoter, did not show the presence of NG2+ cells, but showed a slight increase in CDNF immunoreactivity in GFAP+ cells (Additional file 3: Figure S3, A) when compared to that in intact tissue (Figure 5A). However, this was less than that produced by the hCDNF transfection under control of 3xNBRE promoter (Figure 5C and Additional file 3: Figure S3C), while the transfection of EGFP under 3xNBRE promoter showed similar effects (Additional file 3: Figure S3B) as observed in L30 (Figure 5B). MFI for NG2 (Additional file 3: Figure S3D) showed around 10-fold and 15-fold increase with p3xNBRE-EGFP (30.72 ± 1.82) and with p3xNBRE-hCDNF (45.85 ± 2.74) respectively, when compared to that of pCR3.1-hCDNF (2.65 ± 0.74) transfection. MFI for CDNF (Additional file 3: Figure S3F) showed approximately 2-fold and 4-fold increase with p3xNBRE-EGFP (38.89 ± 2.83) and with p3xNBRE-hCDNF (57.1 ± 2.6) respectively, when compared to that of pCR3.1-hCDNF (22.15 ± 5.62) transfection. Whereas, MFI for GFAP (Additional file 3: Figure S3E) showed no significance at L15FT15 (35.8 ± 1.97) but a significant increase at L15T15 (48.97 ± 1.36), when compared to that at L15OT15 (35.99 ± 3.05). These results suggest that the combination of hCDNF gene and 3xNBRE promoter is responsible for the increased production of endogenous rCDNF by astrocyte-like NG2 cells as this phenomenon is less pronounced with a reporter gene and absent with CMV promoter.

### hCDNF transfection reduces nitrosative stress in the SN

**Figure 6:**
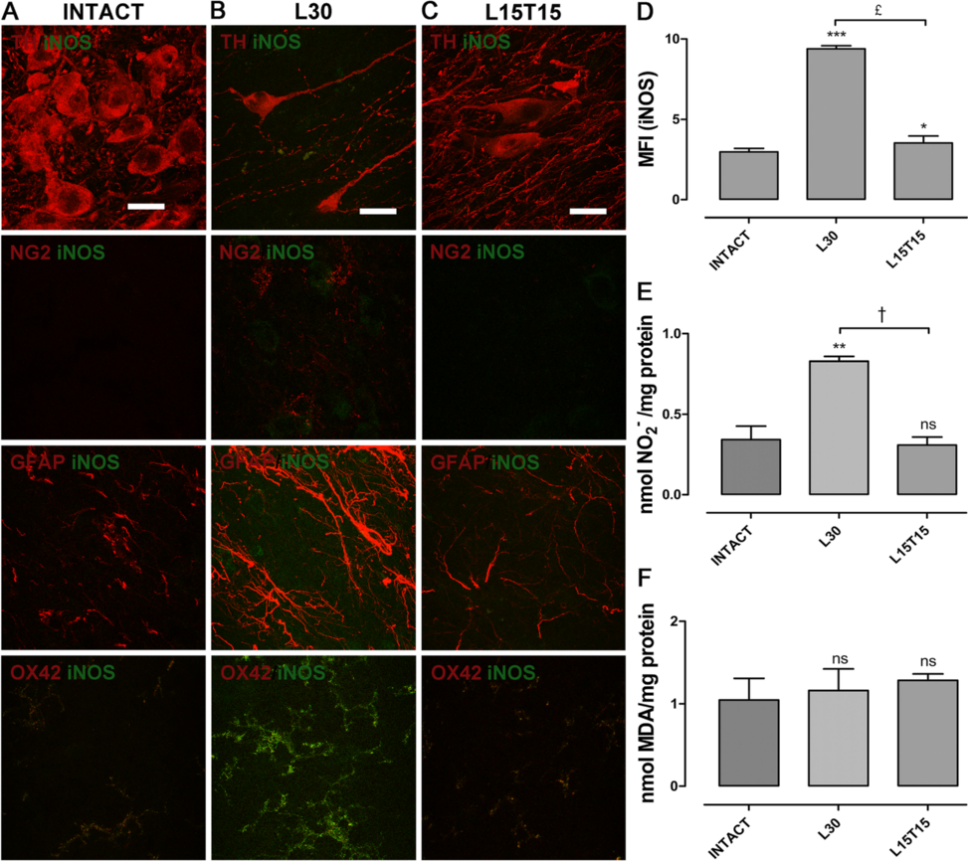
**Nitrosative and oxidative stress markers in the substantia nigra (SN).** Representative merged confocal micrographs showing the presence of inducible nitric oxide synthase (iNOS) + cells (green) with tyrosine hydroxylase (TH) + cells (red) in first row, with NG2+ cells (red) in the second row, with GFAP+ cells (red) in the third row, and with OX42+ cells (red) in the last row in intact **(A)**, in L30 **(B)** and in L15T15 **(C)** conditions. Graphs showing the mean fluorescence intensity (MFI) for iNOS **(D)**, the levels of nitrites **(E)** and malondialdehyde **(F)**. The headings for micrograph panels and x-axis labels for graphs refer to mesencephalon slices that were obtained from different rats, intact, with 30-day 6-hydroxydopamine (6-OHDA) lesion, L30, and 15 days of lesion and 15 days of transfection, L15T15. The scale bars = 20 μm are common for all the micrographs. ns = not significant. *P < 0.05, **P < 0.01, ***P < 0.001 when compared with the intact condition. † = *P* < 0.01, £ = *P* < 0.001 when compared with L30. One-way ANOVA with Newman-Keuls *post hoc* test. n = 4 different rats for each condition. GFAP, glial fibrillary acidic protein; NG2, Neuron/Glial type 2.

We evaluated iNOS immunoreactivity and nitrite levels as markers for nitrosative stress and MDA levels as a marker for oxidative stress (lipoperoxidation). Merged confocal micrographs of double immunofluorescence in intact SNc showed the presence of iNOS immunoreactivity in OX42+ cells and its absence in other glia cells and in TH+ neurons (Figure 6A). In L30, increased iNOS immunoreactivity was found in OX42+ cells (Figure 6B) when compared to that in intact condition (Figure 6A). p3xNBRE-hCDNF transfection reduced both the presence of iNOS and activated glial population-like NG2 cells, astrocytes and microglia in the SNc (Figure 6C). Supporting this, MFI for iNOS (Figure 6D) showed a 3-fold increase with 6-OHDA lesion (9.385 ± 0.191) and reduced almost near to the basal values with hCDNF transfection (3.53 ± 0.44) when compared to intact condition (2.975 ± 0.222). The hCDNF transfection (0.309 ± 0.086 nmol/mg) also significantly decreased the nitrite levels induced (0.83 ± 0.05 nmol/mg) by the lesion (L30) to the basal >values (0.343 ± 0.144 nmol/mg) measured in the intact SN (Figure 6E). No significant difference was found in the levels of lipoperoxide markers at the time points of the study (Figure 6F). These results suggest that transient CDNF expression reduces nitrosative stress in the midbrain.

### hCDNF transfection reduces activated glial cells in the SNc

**Figure 7:**
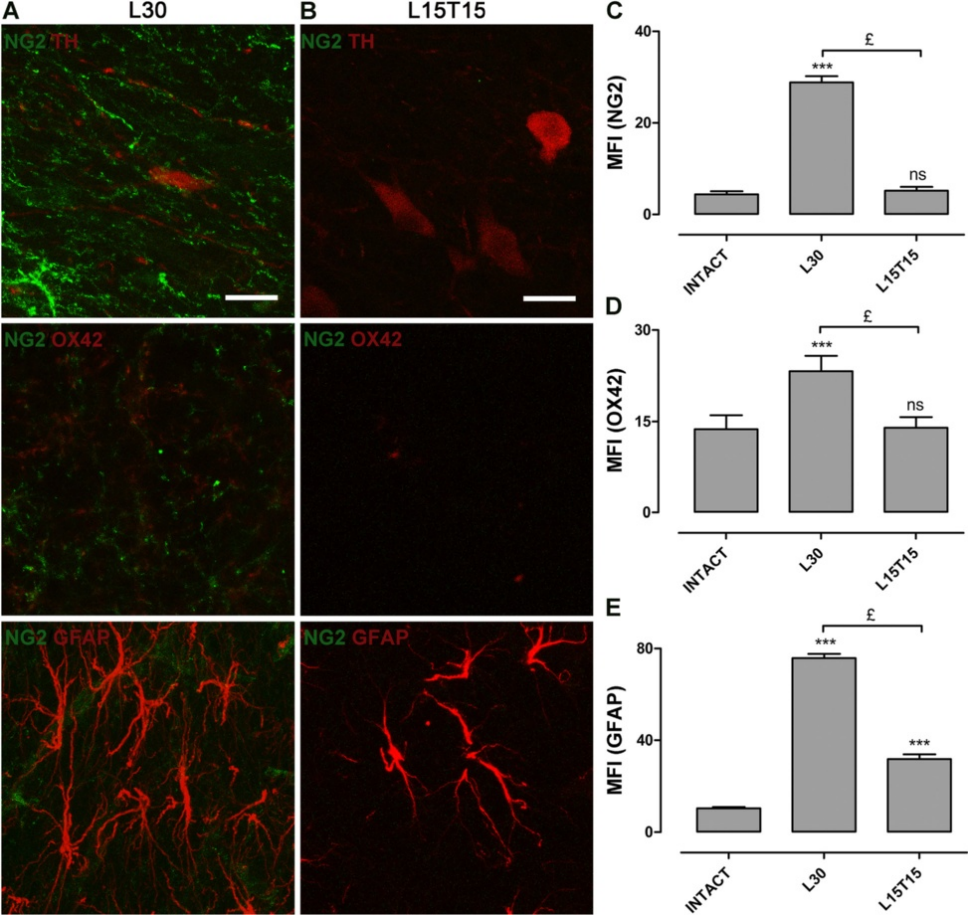
**Presence of glial cells in the substantia nigra pars compacta (SNc).** Representative merged confocal micrographs showing the presence of NG2+ cells (green) with TH+ cells (red, top row), with OX42+ cells (red, middle row), and with GFAP+ cells (red, bottom row) at day 30 after lesion, L30 **(A)** and at day 15 after transfection with 15 days of lesion, L15T15 **(B)**. Graphs showing the mean fluorescence intensity (MFI) for NG2 **(C)**, OX42 **(D)** and GFAP **(E)**. The scale bars = 20 μm are common for all the micrographs. ns = not significant. ***P < 0.001 when compared with the intact condition. £ = *P* < 0.001 when compared with L30. One-way ANOVA with Newman-Keuls *post hoc* test. n = 4 different rats for each condition. GFAP, glial fibrillary acidic protein; NG2, Neuron/Glial type 2.

**Figure 8:**
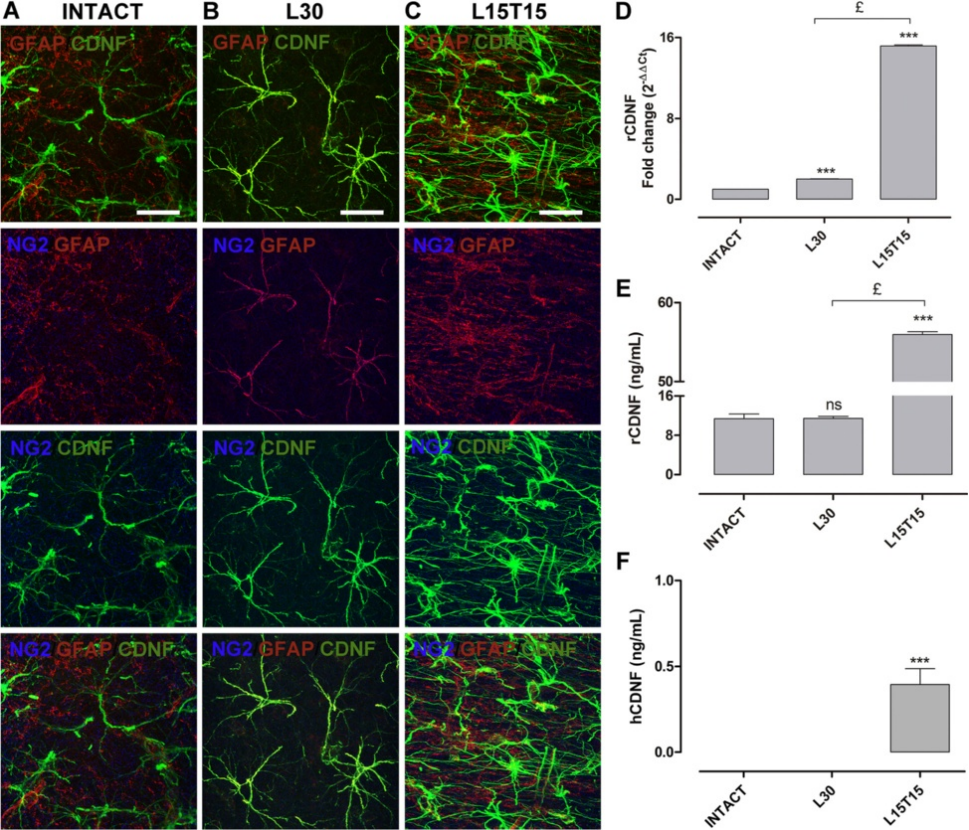
**Expression levels of total cerebral dopamine neurotrophic factor (CDNF) in the striatum.** Representative merged confocal micrographs showing immunoreactivity to CDNF (green), GFAP (red) and NG2 (blue). The triple immune micrographs in the lower row are shown individually in the first three rows with possible combinations of double markers to see more details in intact **(A)**, in L30 **(B)** and in L15T15 **(C)** conditions. Graphs showing rCDNF transcripts by qRT-PCR **(D)** and protein levels of rCDNF **(E)** and hCDNF **(F)** by ELISA. The headings for micrograph panels and x-axis labels for graphs refer to mesencephalon slices that were obtained from different rats, intact, with 30-day 6-hydroxydopamine (6-OHDA) lesion, L30, and 15 days of lesion and 15 days of transfection, L15T15. The scale bars = 20 μm are common for all the micrographs. ns = not significant. ***P < 0.001 when compared with the intact condition. £ = *P* < 0.001 when compared with L30. One-way ANOVA with Newman-Keuls *post hoc* test. n = 4 different rats for each condition. GFAP, glial fibrillary acidic protein; NG2, Neuron/Glial type 2.

As TH+ cells are in the SNc, we used TH immunostaining to locate that region. In intact rat SNc, all the glial cell markers like NG2 (4.135 ± 0.345), OX42 (13.8 ± 0.3) and GFAP (10.44 ± 0.53) were present in low/basal levels as per MFI (Figure 6A; Figure 7C,D,E). In L30 SNc, the double immunofluorescence studies showed the presence (Figure 7A) and 6-fold increase (28.86 ± 1.39) in NG2+ cells (Figure 7C), around 2-fold increase (23.23 ± 2.53) in OX42+ cells (Figure 7D) and 8-fold increase (75.79 ± 1.85) in GFAP+ cells (Figure 7E), indicating the activation of glial cells due to 6-OHDA injection. The transfection of p3xNBRE-hCDNF significantly reduced the presence of NG2+ cells (5.438 ± 0.401) and OX42+ cells (14 ± 2) (Figure 7B) and their respective MFI to the basal levels (Figure 7C and D). The transfection also reduced the branching of GFAP+ cells (Figure 7B) and MFI of GFAP+ cells (31.89 ± 2.05) almost to half (Figure 7E). These results suggest the inactivation of glial cells because of p3xNBRE-hCDNF transfection. No co-localization of markers was observed in either of the experimental conditions. The effect of p3xNBRE-hCDNF plasmid transfection in the SNc was opposite to that in the SNr (Figure 5).

### Expression levels of total CDNF in the striatum

In the intact striatum, GFAP+ and CDNF+ cells were found, but GFAP/CDNF double positive cells and NG2+ cells were absent (Figure 8A). In addition, the morphology of CDNF+ cells was different from that of GFAP+ and NG2+ cells (Figure 8A), suggesting that astrocytes do not produce CDNF in the striatum. In contrast, in L30, extended branching of NG2+ and GFAP+ cells collocating with CDNF+ cells: that is NG2/GFAP/CDNF triple positive cells, were detected (Figure 8B). These suggest that, similar to L30 SNr (Figure 5B), the 6-OHDA injection leads to the activation of glial cells in the striatum. Interestingly, in L15T15, the immunofluorescence analysis showed the absence of NG2/GFAP/CDNF triple positive cells yet there seemed to be an increase in the number CDNF+ cells as compared with the intact and L30 conditions (Figure 8C). Moreover, the morphology of GFAP+ cells after transfection (Figure 8C) appears to be similar as in an intact striatum (Figure 8A), indicating inactivation of those glial cells by overexpressed CDNF. Accordingly, qRT-PCR (Figure 8D) and ELISA (Figure 8E) results showed an increase in the level of rCDNF transcripts (15.16 ± 0.11) and proteins (55.94 ± 0.37 ng/mL) in L15T15 when compared with those in intact (11.37 ± 0.95 ng/mL) and in L30 conditions (2.01 ± 0.02 and 11.41 ± 0.44 ng/mL). hCDNF protein (0.395 ± 0.093 ng/mL) was also detected in the striatum at L15T15 (Figure 8F), suggesting the possible anterograde transport of hCDNF protein from SN to the striatum.

### Levels of Inflammatory cytokines in the nigrostriatal system

**Figure 9:**
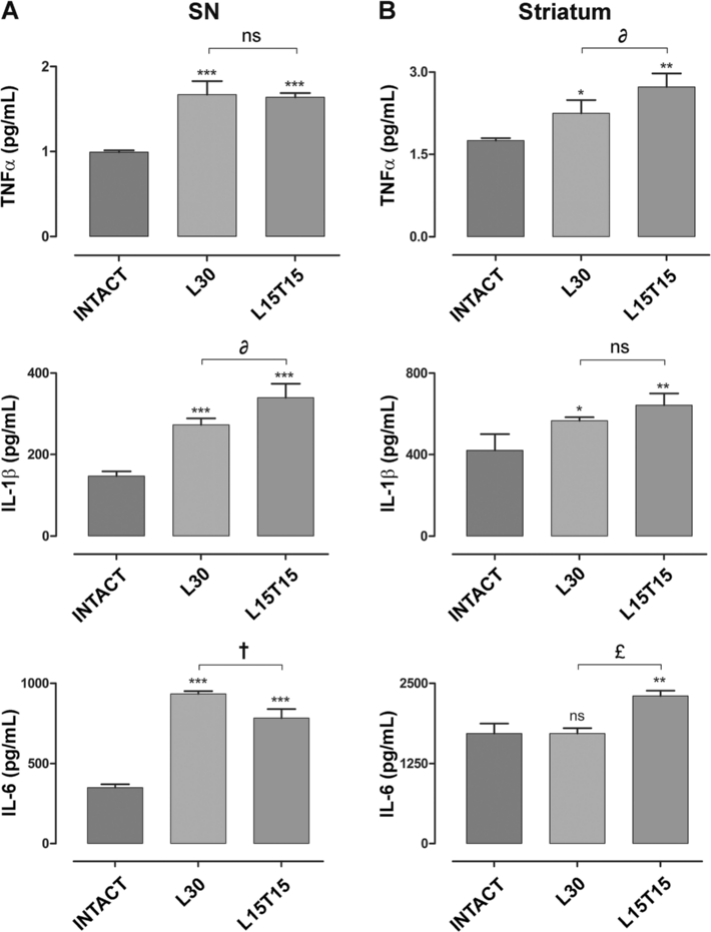
**Protein levels of inflammatory cytokines in the nigrostriatal system.** Graphs showing the protein levels of TNF-α, IL-1β, and IL-6 using indirect ELISA in the substantia nigra (SN) **(A)** and in the striatum **(B)**. ns = not significant, *P < 0.05, **P < 0.01, ***P < 0.001 when compared with the intact condition. ∂ = *P* < 0.05, † = *P* < 0.01, £ = *P* < 0.001 when compared with L30. One-way ANOVA with Newman-Keuls *post hoc* test. n = 4 different rats for each condition.

Basal protein levels of TNF-α (0.991 ± 0.022 pg/mL in SN and 1.751 ± 0.044 pg/mL in striatum), IL-1β (147.8 ± 11.3 pg/mL in SN and 420.2 ± 80.5 pg/mL in striatum) and IL-6 (350.3 ± 20.7 pg/mL in SN and 1,717 ± 155 pg/mL in striatum) were measured by indirect ELISA in an intact rat brain (Figure 9A and B). In L30, TNF-α (1.67 ± 0.16 pg/mL in SN and 2.248 ± 0.242 pg/mL in striatum), IL-1β (272.6 ± 16.3 pg/mL in SN and 566 ± 17 pg/mL in striatum) and IL-6 (933.9 ± 17.8 pg/mL in SN) protein levels increased significantly (except for IL-6 in striatum) when compared to that of intact condition (Figure 9A and B), suggesting that this increase is due to the 6-OHDA lesion. Whereas in L15T15, hCDNF transfection could neither decrease those levels as expected nor could reach basal levels as in intact condition. In L15T15 SN, IL-6 levels (783.3 ± 56.2 pg/mL) decreased, TNF-α levels (1.64 ± 0.05 pg/mL) remained the same but IL-1β levels (339.3 ± 34.6 pg/mL) increased, when compared with those in L30 (Figure 9, A). In L15T15 striatum, both the levels of IL-6 (2,303 ± 83 pg/mL) and TNF-α (2.729 ± 0.246 pg/mL) increased but no significant change was detected in IL-1β (642.2 ± 58.1 pg/mL) levels, when compared with those in L30 (Figure 9B).

## Discussion

The hypothetical view of CDNF as an ER stress response protein [6] opened new research avenues in the field of inflammation. Indeed, the anti-inflammatory role of CDNF was recently shown in cultures of lipopolysaccharide (LPS)-stimulated microglia [16] and tunicamycin-stimulated astrocytes [15]. To the best of our knowledge, the anti-inflammatory role of CDNF has not yet been shown either *in vivo* or in the 6-OHDA model. Therefore, we have focused on anti-neuroinflammatory role of CDNF in the rat 6-OHDA model and found that a single intranigral transient transfection of hCDNF gene at critical period, which is at day 15 after lesion [51], can reduce neuroinflammatory markers. This reduction might be accounted by the overexpression of endogenous rCDNF due to the conversion of NG2 cells to astrocytes and expression of hCDNF. The conversion of NG2 cells to astrocytes was reported in the spinal cord [39,59], in the cerebellum [41] and in the cerebral cortex following a penetrating brain injury [60], but not in other regions of the brain. Supporting this feature, our work showed an apparent conversion of NG2 cells to astrocytes in the SNr of rats induced by the intrastriatal 6-OHDA lesion.

Neuroinflammatory processes and other pathophysiological mechanisms of PD can be reproduced by 6-OHDA in the rat [22,25,26]. Accordingly, a single intrastriatal injection of 6-OHDA in our work also led to the activation of various glial cells, the presence of nitrosative stress markers and increase in the levels of inflammatory cytokines. Interestingly, at day 30 after lesion, we found an increase in the levels of rCDNF transcripts but no significant change at protein level when compared to those in intact SN and striatum. Among several reasons [61-63], changes in the post-transcriptional control [64] or delayed correlation [65] might explain the lack of correlation between mRNA and protein for rCDNF in L30 as those samples were subjected to oxidative stress by 6-OHDA. Supporting this, in L30, triple immunofluorescence results showed conversion of NG2 cells into astrocytes to produce rCDNF in the SNr and in the striatum and these levels seem to be the same as in the intact condition, as were confirmed by ELISA and MFI quantification. These results suggest that the endogenous production of rCDNF in hemiparkinsonian rats was only to maintain the basal levels, which were insufficient to halt the progression of the neuroinflammatory response, as stress markers still appear at this time point.

In our study, intranigral transfection of hCDNF gene under the control of synthetic NBRE (that is Nurr1 dependent) promoter [66-68], was successfully achieved using NTS-polyplex. However, the promoter was inactivated or the expression was shut down in hemiparkinsonian rats presumably due to the depletion in the levels of Nurr1 in degenerated TH+ neurons [69,70] or due to the release of Nurr1 protein from nucleus into cytoplasm [71]. Even though qRT-PCR experiments in the SN show the disappearance of hCDNF transcript at day 3 after p3xNBRE-hCDNF transfection, ELISA experiments show the presence of hCDNF protein in both the SN and striatum even at day 15 after transfection. However, hCDNF protein levels significantly decreased after day 3 post-transfection. As the half-life of hCDNF remains unknown, we can suggest that the levels of hCDNF protein after day 3 post-transfection might correspond to that protein possibly stored in a cell compartment and the reduction might be due to the utilization of protein. This suggestion is supported by the finding that hCDNF protein levels were almost half when compared with pCR3.1-hCDNF transfection at day 15 after transfection, both in the SN and in striatum, as CMV promoter in pCR3.1-hCDNF is still active at day 15 post-transfection (data not shown).

We found basal endogenous levels of rCDNF protein in rat SN and striatum. This rCDNF expression was found in the PBP nucleus and in the SNr, but does not co-localize with TH+ neurons in intact condition, depicting the published result that CDNF does not co-localize with TH+ cells [2]. Interestingly, immunofluorescence results in intact rat SNc/SNr inter-region shows the overlay of rCDNF with few TH+ fibers. However, intranigral transfection in the intact SNc shows the presence of TH/hCDNF double positive cells because of the targeted gene transfection of hCDNF. Surprisingly, we also found induction of rCDNF levels by p3xNBRE-hCDNF transfection and the levels were 2-fold higher than that induced by pCR3.1-hCDNF transfection in hemiparkinsonian rats, and was 4-fold higher than normal/basal levels. The transfection with pCR3.1-hCDNF showed no activation of NG2 cells, yet showed GFAP/CDNF double positive cells, which might be a source for rCDNF. Whereas the presence of NG2/GFAP/CDNF triple positive cells in the SNr in L15T15 is more evident and seems to be more reactive, which is a possible source for rCDNF levels, in accordance with ELISA results. Thus, the presence of TH/CDNF double positive cells in L15T15 may be either due to the presence of hCDNF protein in TH+ neurons or due to the uptake of rCDNF by TH+ fibers that was released from NG2-like astrocytes in the SNr. This entire phenomenon of conversion of one type of glial cell to another type of glial cell to produce neurotrophic factor is apparent in the SNr, and the presence of NG2/GFAP/CDNF triple positive cells might be an indication of a neuroprotective effect.

Our observation that the activation or induction of endogenous rCDNF by transient hCDNF transgene might be either due to homologous recombination or due to the activation of recombinant activator genes (RAGs). From our results in intact and L30 conditions, it is clear that the astrocytes or NG2-like astrocytes are possible sources of rCDNF. Therefore, one possible mechanism behind the induction of rCDNF levels could be that the transgene, hCDNF, directs the conversion of NG2 progenitor cells towards astrocytes. However, as no published reports exist, more in-depth research has to be conducted to identify the mechanism underpinning this phenomenon.

In L15T15, the function and morphology of glial cells in the SNc is completely opposite with those in the SNr. p3XNBRE-hCDNF transfection led to the inactivation of glia cells, decrease of stress markers and absence of NG2/GFAP/CDNF triple positive cells in the SNc, suggesting the anti-inflammatory role of CDNF in this region. The reactive glia found in the SNr in L15T15 might only act as a source of rCDNF but might not participate in inflammation. CDNF reduced the levels of TNF-α, IL-1β and IL-6 in tunicamycin-induced astroglial cell cultures [15], but we observed reduction only in IL-6 levels in SN in *in vivo* 6-OHDA induced PD model. This discrepancy might be explained by the differences in the models used, but also by the fact that we used 6-OHDA instead of tunicamycin, which is known to induce very strong ER stress. The protein levels of inflammatory cytokines increased in both the SN and striatum after 6-OHDA injection and hCDNF transfection remain the same or are slightly increased or decreased depending on the type of cytokine.

Neurotoxic or neurotrophic effects of TNF-α depends on the differential localization of its receptors (TNF-α receptor I or p55 and TNF-α receptor II or p75) in glial and neuronal cells, their mode of activation, and the downstream effectors [72]. Neuroprotective action of TNF-α (both exogenous or that released by astrocytes) was proved when TNF-α treated primary astrocytes showed up-regulation of neural growth factor (NGF), glial cell line-derived neurotrophic factor (GDNF) and brain-derived neurotrophic factor (BDNF) [73]. Signals that activate NF-κB in microglia induce the production of TNF-α. The released TNF-α binds with TNF-α receptor I on neurons and astrocytes to induce neurotrophic factor (NTF) production in response to inflammation, thus promoting neuronal survival [74]. On the other hand, ERK/MAPK (mitogen-activated protein kinase) pathway also regulates TNF-α-induced expression of BDNF via CCAAT/enhancer-binding protein β (C/EBPβ) [75]. Besides TNF-α, IL-1β and IL-6 can also induce activation of C/EBPβ in astrocytes, leading to NTF production [76,77]. Though the pathway behind rCDNF up-regulation in astrocytes is not yet known, available evidence suggests the possible induction of C/EBPβ. TNF-α can also induce its own production by autocrine stimulation, followed by the synthesis of IL-1β and IL-6. Taken together, we propose that either of those increased levels of cytokines leads to CDNF, possibly rCDNF, induction by astrocytes.

In addition, increase in IL-1β levels in L15T15 in SN that can account for the increase of GFAP immunoreactivity. As the association of IL-1β and GFAP expression has shown to be neuroprotective in traumatic injury model [78], we can expect the same effect in the 6-OHDA PD model. Moreover, the increased levels of TNF-α and IL-1β can be beneficial as these cytokines can act as neurotrophic factors in various PD animal models, thus causing recovery from neurodegeneration of the nigrostriatal dopaminergic system [30-32]. IL-6, besides being a pro-inflammatory cytokine, also acts as an anti-inflammatory cytokine [79-82] and plays a major role in neuronal differentiation and survival [83-85]. Considering these, we could propose that IL-6 plays anti-inflammatory and neuroprotective roles in the 6-OHDA dopamine model used in our work as nitrosative stress and glial markers were absent. Increased striatal TNF-α might be adaptive, causing its autocrine stimulation, followed by the synthesis of IL-1β and IL-6 [86]. Based on that possible adaptive response in the striatum, the differences in levels of TNF-α, IL-1β and IL-6 in SN and in striatum at L15T15 might be due to the ability of the striatum to recover from lesion first than the SN. Our results showed inactivation of NG2 glia and astroglia at L15T15 in the striatum but not in SN suggesting that striatum is in next level of recovery from that of SN at that time.

Altogether, the recent findings suggest that the participation of some unknown mediators, probably hCDNF in this case, direct these inflammatory cytokines towards neuroprotection. Reduction of reactive glia and nitrosative stress markers and stimulation of TNF-α, IL-1β and IL-6 leading to CDNF production by astrocytes suggest the anti-inflammatory role of CDNF. It is known that intrastriatal chronic infusion [13] or adeno-associated viral hCDNF gene delivery [11] caused neuronal recovery in a rat 6-OHDA model. Our results provide new insight into the role of CDNF *in vivo* showing for the first time that the acute intranigral human *CDNF* gene transfection reverses at least some aspects of neuroinflammation, possibly as a prelude to its neurotrophic effect.

## Conclusions

The present study is the first report demonstrating the anti-inflammatory effect of CDNF *in vivo* and in the rat 6-OHDA model of PD. Transient transgenic expression of hCDNF together with 6-OHDA lesion controls the activation and conversion of other glial cells, like NG2 and astrocytes, and augments endogenous rCDNF production. In the process of neurodegeneration caused by 6-OHDA, stress, including nitrosative stress, is a robust and first event that is responsible for the apoptotic death of dopaminergic neurons. We demonstrated that CDNF is the neurotrophic factor that directly acts at this level to relieve stress and might prevent the further death of neurons.

## Additional files

Additional file 1: Figure S1. Sequential 1-μm optical sections showing the apparent conversion of a single NG2 cell into an astrocyte cell in L30 SNr. Representative confocal micrographs showing immunoreactivity to NG2 (green) and GFAP (red). NG2/GFAP is the overlay of the micrographs in the same row. The scale bars = 20 μm are common for all the micrographs. Additional file 2: Figure S2. NG2 cells neither convert into microglia cells nor produce CDNF in the SNr. Representative merged confocal micrographs showing immunoreactivity to CDNF (green), OX42 (red), and NG2 (blue). The triple immune micrographs in the lower row are expressed individually in first three rows with possible combinations of double markers to see more details. The headings refer to mesencephalon slices that were obtained from different rats, intact (A), with 30-day 6-OHDA lesion, L30 (B), and 15 days of lesion and 15 days of transfection, L15T15 (C). The scale bars = 20 μm are common for all the micrographs. Additional file 3: Figure S3. CDNF immunoreactivity with astrocytes and/or astrocyte-like NG2 cells in SNr when transfected with different plasmids. Representative merged confocal micrographs showing immunoreactivity to CDNF (green), GFAP (red), and NG2 (blue) in the mesencephalon slices of rats after 15 days of lesion and 15 days of transfection with pCR3.1-hCDNF (A, L15OT15), with p3xNBRE-EGFP (B, L15FT15) and with p3xNBRE-hCDNF (C, L15T15); same as shown in Figure 4, C. The triple immune micrographs in the lower row are expressed individually in first three rows with possible combinations of double markers to see more details. Asterisk represents the cell whose fluorescent markers are individually displayed in the inserts. Graphs showing the mean fluorescence intensity (MFI) for NG2 (D), GFAP (E) and CDNF (F). The scale bars = 20 μm is common for all the micrographs. ns = not significant. ****P* < 0.001 when compared with the intact condition. £ = *P* < 0.001 when compared with L30. One-way ANOVA with Newman-Keuls *post hoc* test. n = 4 different rats for each condition.

## Abbreviations

4-HEA: 4-hydroxyalkanal
6-OHDA: 6- hydroxydopamine
ANOVA: analysis of variance
BDNF: brain-derived neurotrophic factor
bp: base pair
BSA: bovine serum albumin
CDNF: cerebral dopamine neurotrophic factor
C/EBPβ: CCAAT/enhancer-binding protein β
CMV: cytomegalovirus
COX2: cyclooxygenase
EDTA: ethylenediaminetetraacetic acid
EGFP: enhanced green fluorescent protein
EGTA: ethylene glycoltetraacetic acid
ELISA: enzyme-linked immunosorbent assay
ER: endoplasmic reticulum
ERK/MAPK: mitogen-activated protein kinase
GDNF: glial cell line-derived neurotrophic factor
GFAP: glial fibrillary acidic protein
hCDNF: human CDNF
IL: interleukin
iNOS: inducible nitric oxide synthase
LPS: lipopolysaccharide
MANF: mesencephalic astrocyte-derived neurotrophic factor
MDA: malondialdehyde
MFI: mean fluorescence intensity
MPTP: 1-methyl-4-phenyl-1,2,3,6-tetrahydropyridine
NBRE: nerve growth factor responsive element
NF: nuclear factor
NG2: Neuron/Glial type 2
NGF: neural growth factor
NO: nitric oxide
NTF: neurotrophic factor
NTS: neurotensin
OX42: marker for brain microglia
PBP: parabranchial pigmented
PBS: phosphate-buffered saline
PD: Parkinson’s disease
qRT-PCR: quantitative Reverse Transcription- Polymerase Chain Reaction
RAGs: recombinant activator genes
rCDNF: rat CDNF
ROI: region of interest
SN: substantia nigra
SNc: substantia nigra pars compacta
SNr: substantia nigra pars reticulata
SNc/SNr: inter-region between substantia nigra pars compacta and pars reticulata
TH: tyrosine hydroxylase
TNF-α: tumor necrosis factor alpha
