# Supplementary figures and images for: Transient transfection of human CDNF gene reduces the 6-hydroxydopamine-induced neuroinflammation in the rat substantia nigra
Source: J Neuroinflammation. 2014 Dec 16;11:209. doi: 10.1186/s12974-014-0209-0 (PMC4275959; doi:10.1186/s12974-014-0209-0)

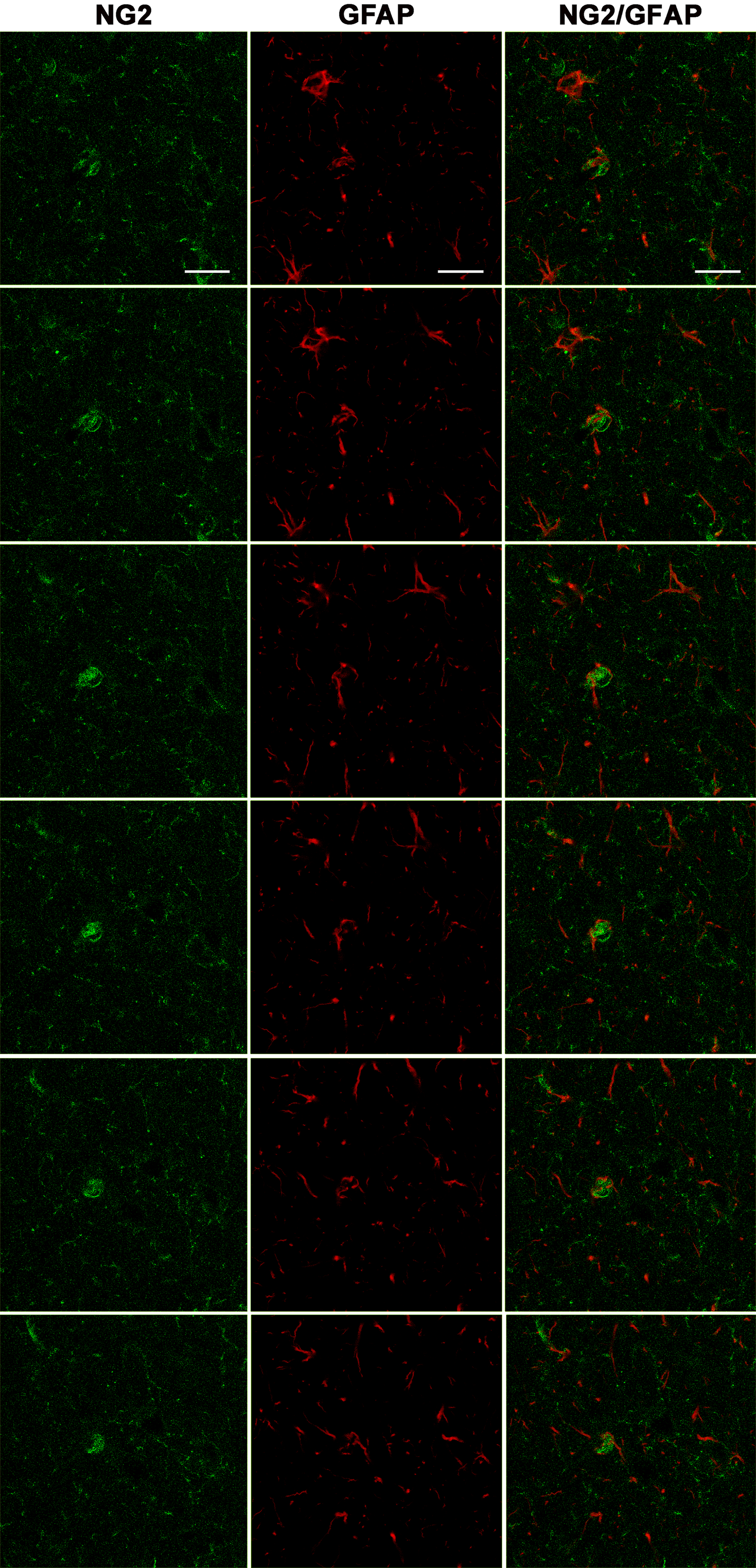

Supplement: Additional file 1: Figure S1. — Sequential 1-μm optical sections showing the apparent conversion of a single NG2 cell into an astrocyte cell in L30 SNr. Representative confocal micrographs showing immunoreactivity to NG2 (green) and GFAP (red). NG2/GFAP is the overlay of the micrographs in the same row. The scale bars = 20 μm are common for all the micrographs. [file 12974_2014_209_MOESM1_ESM.tiff]

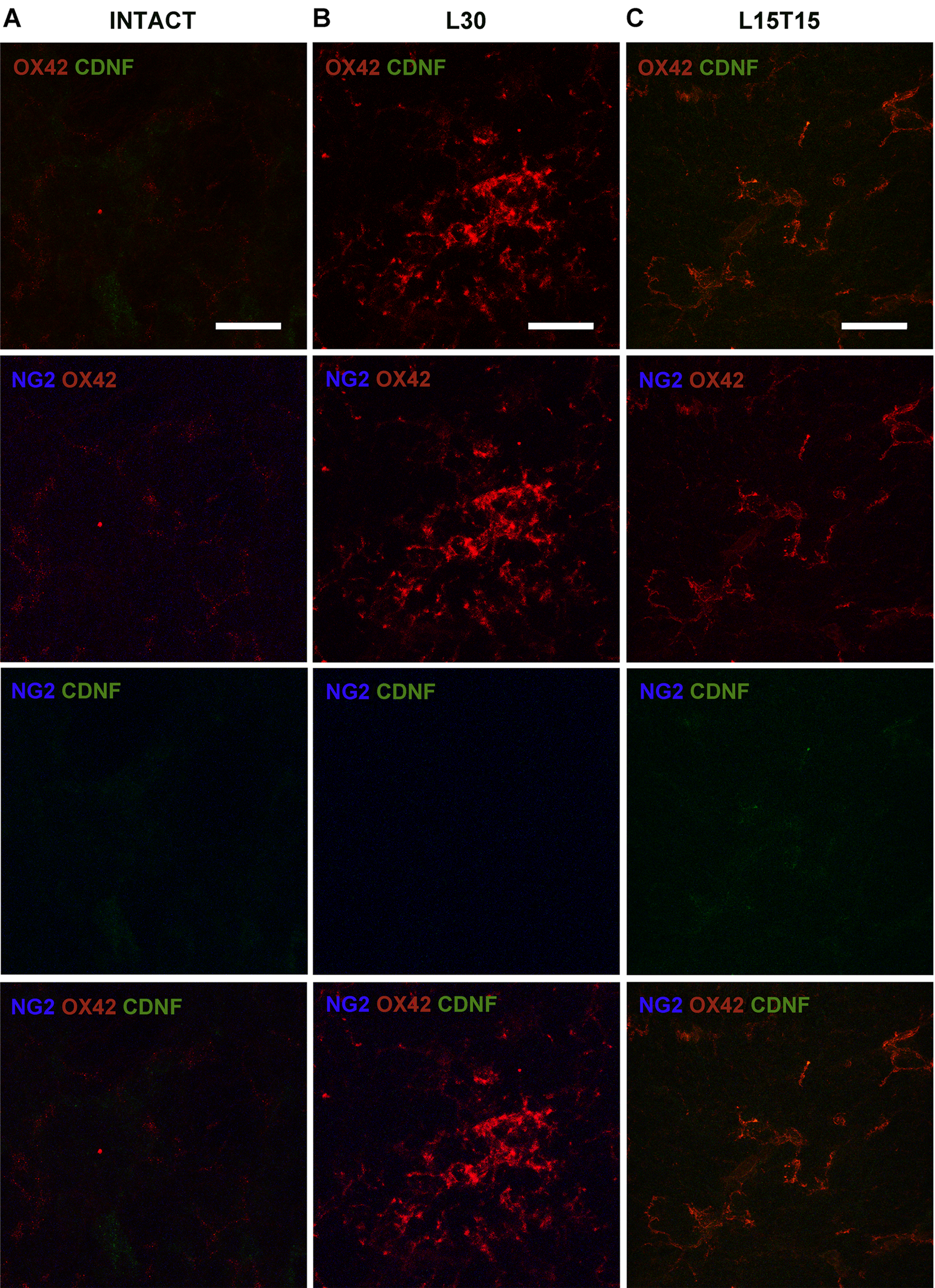

Supplement: Additional file 2: Figure S2. — NG2 cells neither convert into microglia cells nor produce CDNF in the SNr. Representative merged confocal micrographs showing immunoreactivity to CDNF (green), OX42 (red), and NG2 (blue). The triple immune micrographs in the lower row are expressed individually in first three rows with possible combinations of double markers to see more details. The headings refer to mesencephalon slices that were obtained from different rats, intact (A), with 30-day 6-OHDA lesion, L30 (B), and 15 days of lesion and 15 days of transfection, L15T15 (C). The scale bars = 20 μm are common for all the micrographs. [file 12974_2014_209_MOESM2_ESM.tiff]

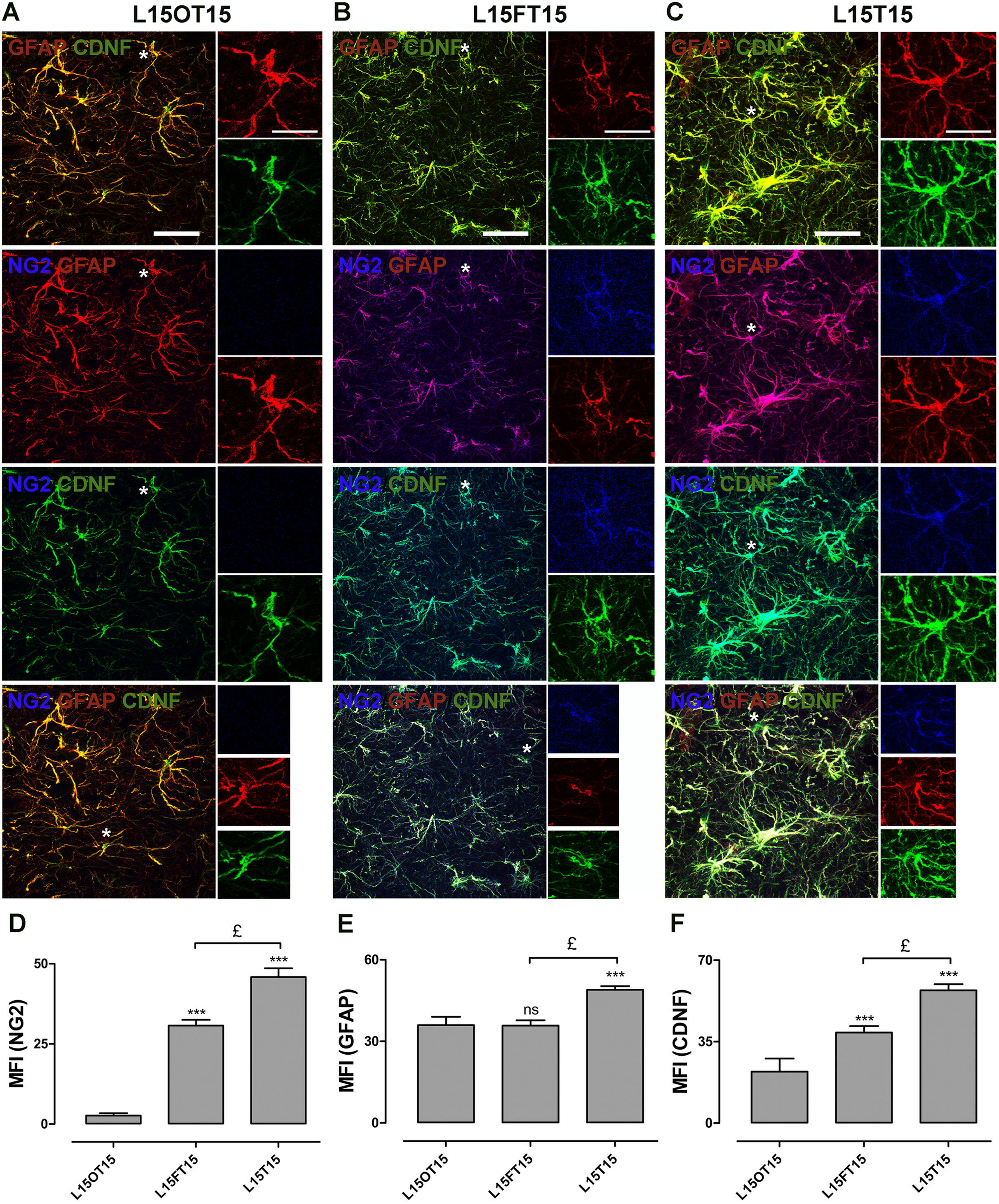

Supplement: Additional file 3: Figure S3. — CDNF immunoreactivity with astrocytes and/or astrocyte-like NG2 cells in SNr when transfected with different plasmids. Representative merged confocal micrographs showing immunoreactivity to CDNF (green), GFAP (red), and NG2 (blue) in the mesencephalon slices of rats after 15 days of lesion and 15 days of transfection with pCR3.1-hCDNF (A, L15OT15), with p3xNBRE-EGFP (B, L15FT15) and with p3xNBRE-hCDNF (C, L15T15); same as shown in Figure 4, C. The triple immune micrographs in the lower row are expressed individually in first three rows with possible combinations of double markers to see more details. Asterisk represents the cell whose fluorescent markers are individually displayed in the inserts. Graphs showing the mean fluorescence intensity (MFI) for NG2 (D), GFAP (E) and CDNF (F). The scale bars = 20 μm is common for all the micrographs. ns = not significant. ***P < 0.001 when compared with the intact condition. £ = P < 0.001 when compared with L30. One-way ANOVA with Newman-Keuls post hoc test. n = 4 different rats for each condition. [file 12974_2014_209_MOESM3_ESM.tiff]
